# Beneficial microbes going underground of root immunity

**DOI:** 10.1111/pce.13632

**Published:** 2019-08-05

**Authors:** Ke Yu, Corné M.J. Pieterse, Peter A.H.M. Bakker, Roeland L. Berendsen

**Affiliations:** ^1^ Plant‐Microbe Interactions, Institute of Environmental Biology, Department of Biology, Science4Life Utrecht University Utrecht 3508 TB The Netherlands

**Keywords:** host immune evasion, plant immunity, plant microbiome, rhizosphere, soil microbiology

## Abstract

Plant roots interact with an enormous diversity of commensal, mutualistic, and pathogenic microbes, which poses a big challenge to roots to distinguish beneficial microbes from harmful ones. Plants can effectively ward off pathogens following immune recognition of conserved microbe‐associated molecular patterns (MAMPs). However, such immune elicitors are essentially not different from those of neutral and beneficial microbes that are abundantly present in the root microbiome. Recent studies indicate that the plant immune system plays an active role in influencing rhizosphere microbiome composition. Moreover, it has become increasingly clear that root‐invading beneficial microbes, including rhizobia and arbuscular mycorrhiza, evade or suppress host immunity to establish a mutualistic relationship with their host. Evidence is accumulating that many free‐living rhizosphere microbiota members can suppress root immune responses, highlighting root immune suppression as an important function of the root microbiome. Thus, the gate keeping functions of the plant immune system are not restricted to warding off root‐invading pathogens but also extend to rhizosphere microbiota, likely to promote colonization by beneficial microbes and prevent growth‐defense tradeoffs triggered by the MAMP‐rich rhizosphere environment.

## Abstract 2

Plant roots interact with an enormous diversity of soil‐borne microbes, which poses a big challenge to roots to distinguish friends from foes. Here, we review current knowledge on how beneficial microbes in the root microbiome actively interfere with the root immune system to establish a mutually beneficial relationship with their host plant.

## 1 THE BELOWGROUND PLANT MICROBIOME

Soils are among the most dense and diverse microbial habitats found on our planet (Fierer & Jackson, 2006). Growing in soil, plant roots intimately interact with this plethora of microorganisms. The complex interactions between the roots and their associated microbiomes are important determinants of plant health (Berendsen, Pieterse, & Bakker, 2012; Mauchline & Malone, 2017; Raaijmakers & Mazzola, 2016; Schlaeppi & Bulgarelli, 2015). Soil‐borne pathogens reduce plant growth, whereas plants can also form associations with microbes that promote plant growth. Such plant‐beneficial microbes can assist the plant with the uptake of nutrients or by enhancing stress tolerance (Pieterse, De Jonge, & Berendsen, 2016; Van der Heijden, Bardgett, & Van Straalen, 2008). Moreover, beneficial microbes can protect plants against pathogens, through antagonism and competition or by stimulating the plant's immune system (Berendsen et al., 2012; Bulgarelli, Schlaeppi, Spaepen, Ver Loren van Themaat, & Schulze‐Lefert, 2013; Pieterse et al., 2014). Well‐studied examples of beneficial microbes include rhizobial bacteria living in symbiosis with legumes and mycorrhizal fungi associated with most terrestrial plants, but there are many other free‐living plant growth‐promoting rhizobacteria (PGPR) and fungi (PGPF) described that benefit a wide range of plant species (Berendsen et al., 2012; Pieterse et al., 2014). Like pathogens, beneficial microbes are confronted with the plant immune system, and they are becoming more and more evident that beneficial microbes similarly need to evade or suppress root immune responses in order to establish a mutualistic relationship with their host (Zamioudis & Pieterse, 2012). This is particularly apparent for endophytes that live inside the plant and are therefore directly exposed to the host immune system (Liu, Carvalhais, Crawford et al., 2017). However, evidence is accumulating that also non‐invasive, free‐living root microbiota members interfere with the host immune system. Here, we review current knowledge on the interplay between beneficial microbes and the plant immune system and how this results in mutual growth or health benefits for the interaction partners.

## 2 IMMUNE SIGNALLING IN ROOTS

Plants have evolved a sophisticated immune system to detect and respond to potential invaders (Cook, Mesarich, & Thomma, 2015; Jones & Dangl, 2006). In plants, cell surface‐localized pattern recognition receptors (PRRs) can detect surrounding microbes by recognizing microbe‐associated molecular patterns (MAMPs), which are generally conserved molecules shared by a wide range of microbes (Boller & Felix, 2009). In the past two decades, numerous MAMPs, such as flagellin, elongation factor Tu (EF‐Tu), cold‐shock protein (CSP), lipopolysaccharide (LPS), chitin, elicitin, and Nep1‐like protein, have been characterized in various plant pathosystems together with their cognate PRRs (Boutrot & Zipfel, 2017). Despite recognizing specific MAMPs, diverse PRRs have been shown to activate convergent cellular immune signalling pathways. Upon MAMP recognition, PRRs recruit regulatory receptor kinases to form PRR complexes that activate a multilayered immune signalling cascade through receptor‐like cytoplasmic kinases (Macho & Zipfel, 2014). The activated immune signalling events, known as MAMP‐triggered immunity (MTI), function in the elimination of potential pathogenic infections (Couto & Zipfel, 2016; Macho & Zipfel, 2014). Ion (H^+^ and Ca^2+^) fluxes and transient bursts of reactive oxygen species (ROS) are two typical cellular responses happening within minutes after immune signalling activation (Boller & Felix, 2009; Yu, Feng, He, & Shan, 2017). Immune signalling is transduced through activation of Ca^2+^‐dependent protein kinase (CDPK) and mitogen‐activated protein kinase (MAPK) cascades, which trigger downstream transcriptional regulation of defence‐related genes, inter alia leading to callose deposition, antimicrobial compounds accumulation, and defence hormone regulation (Boller & Felix, 2009; Couto & Zipfel, 2016; Yu et al., 2017). Plant hormones act as central modulators of many components in the immune signalling network. Two major defence hormones, salicylic acid (SA) and jasmonic acid (JA), form a complex regulatory network to fine‐tune plant immune homeostasis. Other hormones such as auxin, ethylene, abscisic acid, cytokinins, brassinosteroids, and gibberellin also interact with the SA‐ and JA‐regulated defence pathway, together orchestrating the immune signalling network (Pieterse, Van der Does, Zamioudis, Leon‐Reyes, & Van Wees, 2012).

Our knowledge of plant immune signalling mainly comes from studies on interactions between microbes and aboveground plant parts. However, plant roots are also capable of mounting strong immune responses upon PRR‐mediated MAMP recognition, including callose deposition, camalexin biosynthesis, and defence‐related gene activation (Beck et al., 2014; Millet et al., 2010; Stringlis, Proietti, et al., 2018; Wyrsch, Dominguez‐Ferreras, Geldner, & Boller, 2015). Intriguingly, beneficial microbes possess immunogenic MAMPs that are very similar to those of pathogens (Jacobs et al., 2011; Lopez‐Gomez, Sandal, Stougaard, & Boller, 2012; Millet et al., 2010; Pel & Pieterse, 2013; Stringlis, Proietti, et al., 2018). During their initial contact with roots, beneficial microbes are recognized by plant PRRs, activating immune signalling. Root immune activation by beneficial microbes was observed in many root–microbe associations. For example, *Bradyrhizobium japonicum* strongly induces defence‐related gene expression at the early stage of infection in soybean root hair cells (Libault et al., 2010). Also, the arbuscular mycorrhizal fungus *Glomus versiforme* induces a substantial set of defence‐ and stress‐related genes during the initial contact with *Medicago truncatula* (Liu et al., 2003). Similarly, the cellular components of two PGPRs, *Pseudomonas simiae* WCS417 (hereafter, WCS417) and *Pseudomonas capeferrum* WCS358 (hereafter, WCS358), trigger immune responses in *Arabidopsis* roots and tobacco cells, including ROS production, MAMP‐responsive gene expression, and callose deposition (Millet et al., 2010; Stringlis, Proietti, et al., 2018; Van Loon, Bakker, Van der Heijdt, Wendehenne, & Pugin, 2008). Moreover, *Piriformospora indica* has significantly reduced colonization of the roots of an MAMP‐hyper‐responsive *Arabidopsis* mutant *pub22/23/24*, indicating that this PGPF can be recognized by plant PRRs (Jacobs et al., 2011). Together, these studies show that root immune responses are indeed also induced by beneficial microbes. However, this induction appears to be mostly restricted to the early stages of these beneficial associations, suggesting an active interference of root immunity by beneficial microbes.

## 3 MICROBIAL EVASION AND SUPPRESSION OF PRR SIGNALLING IN ROOTS

**Figure 1:**
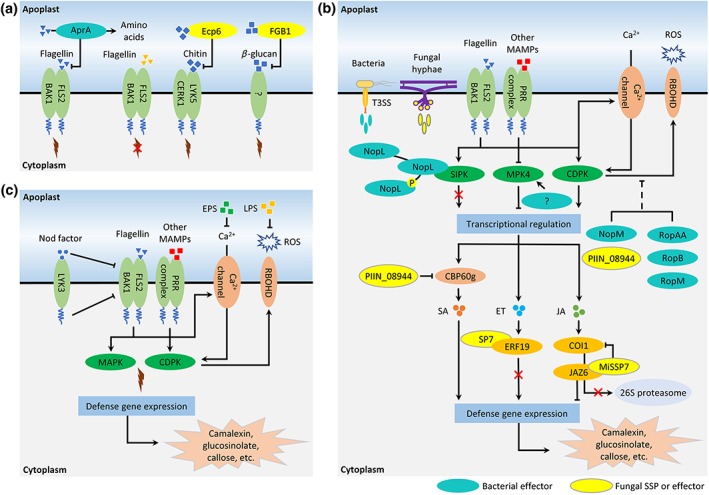
Schematic overview of beneficial microbes that evade or suppress root immune responses as described in the main text. (a) Beneficial microbes can evade PRR recognition by either evolving divergent MAMPs or masking the presence of excessive MAMPs. (b) Beneficial microbes can interfere with different host immune signalling components by secreting effectors. (c) Symbiosis‐related molecules produced by symbiotic microbes can suppress root immunity [Colour figure can be viewed at http://wileyonlinelibrary.com]

To promote infection, successful plant pathogens utilize virulence factors that interfere with immune signalling events (Couto & Zipfel, 2016). Such virulence factors have been well documented for many plant pathosystems (Couto & Zipfel, 2016; Lo Presti et al., 2015; Macho & Zipfel, 2015; Xin & He, 2013). Because MAMPs are conserved molecules shared by many members throughout the microbial kingdoms, it is likely that all plant‐colonizing microbes, pathogens and mutualists alike, have evolved strategies to deal with host immune activation under the selection pressure posed by plant PRRs during the coevolution process. So far, several mechanisms by which beneficial microbes avoid activation of the plant immune system have been described (Figure 1). In the following sections, we will present examples of beneficial microbes that either evade PRR‐mediated immune recognition or interfere with the subsequent immune signalling process.

### 3.1 Evasion of apoplastic recognition

#### 3.1.1 Evolution of divergent MAMPs

Flagellin monomers are the building blocks of bacterial flagella, an essential organelle responsible for bacterial motility (Pel & Pieterse, 2013; Rossez, Wolfson, Holmes, Gally, & Holden, 2015). The *Arabidopsis* PRR FLS2 can recognize flagellin by binding the immunogenic flg22 epitope, a highly conserved sequence of 22 amino acids at the N‐terminus of the protein (Felix, Duran, Volko, & Boller, 1999; Gomez‐Gomez & Boller, 2000; Sun et al., 2013). This happens only when plant glycosidases have degraded the glycosylations that shield the peptide and thus make flg22 available for recognition (Buscaill et al., 2019). Driven by coevolution, variation of flagellin sequences enables certain pathogenic bacteria to evade immune recognition (Rossez et al., 2015). Interestingly, flagellin sequences of the atmospheric nitrogen‐fixing symbiont *Sinorhizobium meliloti* exhibit an exceptional divergence in this region, resulting in a complete abolishment of immune activation in *Arabidopsis* (Felix et al., 1999). Also in *Lotus japonicus*, purified flagellin from the symbiont *Mesorhizobium loti* failed to activate immune responses (Figure 1a), whereas the commonly used flg22 epitope of *Pseudomonas aeruginosa* induced typical immune responses, such as ethylene production, MAPK activation, and defence‐related gene expression, indicating that the FLS2 receptor homolog in *L. japonicus* is fully functional (Lopez‐Gomez et al., 2012). Similar observations were made in the beneficial association formed by an endophytic PGPR *Burkholderia phytofirmans* and grapevine (Figure 1a). The grapevine FLS2 receptor differentially recognizes flg22 epitopes derived from beneficial *B. phytofirmans*, initiating significantly reduced immune responses compared with the immune responses induced by the flg22 epitopes derived from the pathogenic bacteria *P. aeruginosa* and *Xanthomonas campestris* (Trda et al., 2014). It is known that *Arabidopsis* can recognize the bacterial MAMPs flagellin and EF‐Tu through the cognate PRRs FLS2 and EFR, whereas this plant species is unresponsive to CSP for which it misses the cognate PRR CORE (Gomez‐Gomez & Boller, 2000; Wang et al., 2016; Zipfel et al., 2006). This possibly explains why metagenomes of healthy *Arabidopsis* root microbiota possess a fourfold to tenfold higher percentage of genes encoding the nonimmunogenic epitope of CSP than of genes encoding the immunogenic epitopes of flagellin and EF‐Tu (Hacquard, Spaepen, Garrido‐Oter, & Schulze‐Lefert, 2017). These results together suggest that plants can actively select the members of their microbiome through the function of PRRs, whereas many soil‐borne microbes have evolved to evade PRR‐mediated immune recognition in order to form an association with their host plants.

#### 3.1.2 Hiding excessive MAMPs

Both pathogenic and beneficial microbes have been found to conceal the presence of certain immunogenic MAMPs. AprA is an extracellular alkaline protease that is secreted by the pathogenic bacteria *P. aeruginosa* and *Pseudomonas syringae*. AprA degrades flagellin monomers, thereby preventing immune recognition of flagellin both in mammals and plants (Bardoel et al., 2011; Pel et al., 2014; Figure 1a). AprA homologs are present in a wide range of bacterial species, among which many are plant‐beneficial, including nitrogen‐fixing rhizobia and plant growth‐promoting pseudomonads (Pel et al., 2014). Chitin is a major component of fungal cell walls, which triggers immune responses upon recognition by its cognate PRRs in various hosts (Cao et al., 2014; Shimizu et al., 2010). However, the fungal pathogen *Cladosporium fulvum* secretes two lectin‐type chitin‐binding effectors, Avr4 and Ecp6, that strengthen fungal cell walls against hydrolysis by plant‐derived chitinases and prevent immune recognition of chitin by the plant PRR CERK1 (De Jonge et al., 2010; Van den Burg, Harrison, Joosten, Vervoort, & De Wit, 2006; Figure 1a). Ecp6‐like proteins were also found in many other fungal species, including the biological control agent *Chaetomium globosum* (Bolton et al., 2008). Although the role of AprA and Ecp6 homologs in prevention of immune recognition of beneficial microbes still requires confirmation, similar mechanisms were revealed in the PGPF *P. indica* during colonization of both barley and *Arabidopsis* roots (Figure 1). β‐glucan, a fungal cell wall component, can trigger immune responses upon recognition by an uncharacterized PRR complex (Wawra et al., 2016). *P. indica* produces a small secreted protein (SSP) called fungal‐specific β‐glucan‐binding lectin (FGB1), which potentially increases fungal cell wall integrity and interferes with host immune recognition through its high affinity with β‐glucan (Wawra et al., 2016; Figure 1a). This suggests that, like pathogens, beneficial microbes also evolved ways to obscure their most excessively present MAMPs to prevent recognition by their host plants and avoid activation of the plant immune system.

### 3.2 Suppression of cytoplasmic immune signalling

Pathogens can deliver effector proteins into plant cells using, for example, bacterial type III secretion system (T3SS) or the infection structures of fungi and oomycetes. These effector proteins can target various components of plant immune signalling initiated upon MAMP recognition (Couto & Zipfel, 2016; Dodds & Rathjen, 2010; Win et al., 2012). Like plant pathogens, beneficial microbes also utilize a diverse range of effector proteins to suppress plant immune activation. The T3SS is found in the genomes of many plant beneficial rhizobacteria including rhizobia and pseudomonads (Berendsen et al., 2015; Deakin & Broughton, 2009; Loper et al., 2012; Stringlis, Zamioudis, Berendsen, Bakker, & Pieterse, 2019; Figure 1b). Likewise, genomes of many beneficial fungi, such as *Laccaria bicolor* and *P. indica*, possess a substantial set of genes encoding effector‐type small secreted proteins (SSPs) that are highly expressed during root colonization (Martin et al., 2008; Zuccaro et al., 2011; Figure 1b). Moreover, metagenomes of the root microbiomes of cucumber, wheat, citrus, and barley display a significant enrichment of T3SS genes at a community level that are possibly involved in suppression of root immune responses (Bulgarelli et al., 2015; Ofek‐Lalzar et al., 2014; Zhang et al., 2017). This suggests that also non‐pathogenic members of the root microbiome actively interfere with plant immune signalling through the delivery of immune‐suppressive effector molecules, but the research field on this topic is still in its infancy.

#### 3.2.1 Eliminating ROS burst

Rhizobial T3SS effectors are designated nodulation outer proteins (Nops) and mostly function in the regulation of nodulation or determination of host specificity (Miwa & Okazaki, 2017). Nonetheless, several Nop effectors have a direct role in suppressing PRR‐mediated immune signalling (Figure 1b). ROS are generated within minutes upon PRR‐mediated MAMP recognition and function as important signalling molecules in plant immunity (Kimura, Waszczak, Hunter, & Wrzaczek, 2017; Torres, Jones, & Dangl, 2006). NopM, an effector secreted by *Sinorhizobium* sp. strain NGR234, is an E3 ubiquitin ligase that is essential for normal nodulation in *Lablab purpureus* (Xin et al., 2012). Interestingly, in *Nicotiana benthamiana*, NopM was found to suppress flg22‐induced ROS bursts (Xin et al., 2012; Figure 1b). Also, the genome of the biological control strain *Pseudomonas brassicacearum* Q8r1‐96 contains orthologs of pathogen effector genes, and these are expressed in the rhizosphere (Almario et al., 2017; Mavrodi et al., 2011). Infiltration of each of these effectors suppressed flg22‐induced ROS production in *Nicotiana tabacum* (Figure 1b), although deletion of the effector genes did not affect bacterial rhizosphere competence (Mavrodi et al., 2011).

Another example is the *P. indica* effector PIIN_08944, which promotes fungal colonization of the roots of *Arabidopsis* and barley (Akum, Steinbrenner, Biedenkopf, Imani, & Kogel, 2015). Overexpression of *PIIN_08944* significantly reduced the flg22/chitin‐induced ROS burst in barley (Figure 1b), however, not in *Arabidopsis* (Akum et al., 2015). These examples suggest that effectors delivered by beneficial microbes can efficiently perturb plant immunity by eliminating the transient ROS burst.

#### 3.2.2 Targeting MAPK cascades

MAPK cascades control numerous downstream immune signalling events and are targets of many pathogen effectors (Meng & Zhang, 2013). Unsurprisingly, MAPK cascades seem to be targeted by beneficial microbes as well (Figure 1b). For example, *in‐planta* expression of the effector NopL from the *Sinorhizobium* sp. strain NGR234 suppresses the expression of pathogenesis‐related defence proteins in *N. tabacum* and *L. japonicus*. Moreover, NopL prevents cell death induced by overexpression of the MAPK‐encoding gene *SIPK* (Bartsev et al., 2004; Ge et al., 2016; Zhang, Chen, Lu, Xie, & Staehelin, 2011). By mimicking an MAPK phosphorylation substrate, NopL is multiply phosphorylated by SIPK in the nucleus (Figure 1b). Likely, this inhibits the phosphorylation of other natural MAPK substrates that regulate the expression of defence‐related genes, which ultimately results in an interruption of immune signalling (Ge et al., 2016; Zhang et al., 2011).

Another example of MAPK cascade suppression has been described in soybean, in which GmMPK4 regulates the expression of genes encoding WRKY, MYB, and bHLH transcription factors and prevents defence‐related gene expression (Liu et al., 2011). GmMPK4 is significantly induced at the early stage of infection, when soybean is inoculated with *Sinorhizobium fredii* HH103 (Figure 1b) but not when the T3SS of this rhizobial strain was inactivated (Jimenez‐Guerrero et al., 2015). By interfering with host MAPK cascades, beneficial microbes can thus block immune signal transduction and activation of downstream immune responses.

#### 3.2.3 Modulation of hormonal signalling

Because plants use hormones to fine‐tune immune homeostasis during plant–microbe interactions (Pieterse et al., 2012), many pathogens evolved effectors that hijack hormonal signalling pathways (Kazan & Lyons, 2014). Similarly, beneficial microbes have been found to target hormonal signalling pathways to suppress root immune responses and promote their association with the host plant (Figure 1b). For example, the arbuscular mycorrhizal fungus *Rhizophagus irregularis* secrets the effector SP7, which directly interacts with the JA/ethylene inducible‐ERF19 transcription factor and prevents the expression of EFR19‐activated defence‐related genes in *M. truncatula* roots (Kloppholz, Kuhn, & Requena, 2011, Figure 1b). In the nonhost *Arabidopsis*, *R. irregularis* activates rather than suppresses host immunity (Fernández et al., 2019), suggesting that this immune evasion mechanisms fails in nonhost plants. The ectomycorrhizal fungus *L. bicolor* secrets the MiSSP7 effector to promote the establishment of a mutualistic association with *Populus* (Plett et al., 2014). MiSSP7 prevents JA‐induced degradation of JAZ6, a protein functioning as a negative regulator of JA‐induced genes, thus suppressing JA‐mediated transcriptional activation of immune responses such as cell wall modifications (Plett et al., 2014; Figure 1b). The PGPF *P. indica* suppresses flg22‐induced root immune responses in *Arabidopsis* (Jacobs et al., 2011). However, the immunosuppressive phenotype is compromised in JA‐signalling deficient mutants *jar1‐1* and *jin1‐1* (Jacobs et al., 2011). Moreover, the PIIN_08944 effector of *P. indica* has been shown to suppress the expression of flg22‐induced SA marker gene *CBP60g* in *Arabidopsis* (Figure 1b), which encodes a transcription factor that is required for the production of SA by regulating the key biosynthetic enzyme isochorismate synthase 1 (Akum et al., 2015; Wang et al., 2011; Zhang et al., 2010). Similarly, the PGPR *Bacillus subtilis* FB17 can suppress early flg22‐induced root immune responses in *Arabidopsis* by releasing an unidentified low‐molecular weight component, and this immune suppression phenotype also requires functional JA signalling components JAR1, JIN1, and MYC2 (Lakshmanan et al., 2012).

Collectively, these findings provide evidence that beneficial microbes suppress root immune responses through various immune suppressors such as effector proteins, targeting multiple signalling components that are initiated upon MAMP recognition.

### 3.3 Interplay between immunity and symbiosis signalling

In addition to MAMPs, symbiotic microbes also produce different types of symbiosis‐related molecules, which can be recognized by host symbiotic receptors and initiate symbiosis signalling (Zipfel & Oldroyd, 2017). Many of these symbiotic molecules are very similar to MAMPs. For example, rhizobial Nod factors and fungal Myc factors can be perceived by cognate receptors in host plants and initiate rhizobial or arbuscular mycorrhizal symbiotic process (Zipfel & Oldroyd, 2017). Both Nod factors and Myc factors are lipochitin oligosaccharides that are structurally similar to the well‐studied MAMPs chitin and peptidoglycans (Liang et al., 2014). However, it was shown that legume roots can separately recognize the fungal MAMP chitin (immunogenic signal) and Nod factors (symbiotic signal) through two sets of distinct LysM PRRs (Bozsoki et al., 2017). Interestingly, many symbiotic molecules derived from beneficial microbes seem to suppress MAMP‐triggered root immune responses (Figure 1c). Nod factors of *B. japonicum* can strongly suppress immune responses induced by various MAMPs in both soybean and *Arabidopsis*, likely as a result of significantly reduced protein levels of cognate PRRs on the cell membrane (Liang et al., 2013; Figure 1c). Surprisingly, Nod factors can still suppress immune responses in soybean mutants lacking Nod factor receptors but not in an *Arabidopsis* mutant lacking the LysM receptor LYK3 (Liang et al., 2013).

Similarly, rhizobial LPS is required for the establishment of successful symbiosis in legume plants (Gibson, Kobayashi, & Walker, 2008). However, LPS of *S. meliloti* was found to suppress not only early immune responses such as ROS burst but also late defence‐related transcriptional reprogramming in *M. truncatula* (Figure 1c), despite inducing a strong ROS burst in the nonhost *N. tabacum* (Scheidle, Groß, & Niehaus, 2005; Tellstrom et al., 2007). A recent study has shown that EPR3‐mediated recognition of compatible exopolysaccharides (EPS) in *L. japonicus* is crucial in controlling successful entry by *M. loti* (Kawaharada et al., 2015). In addition to its role in symbiosis, EPS derived from *S. meliloti* can block flg22‐induced calcium influx through chelation with calcium ions (Figure 1c), thus suppressing downstream immune responses (Aslam et al., 2008).

Moreover, *Medicago truncatula* mutants impaired in the production of the receptor‐like kinase *LYK9* were less colonized by arbuscular mycorrhiza *Rhizophagus irregularis*, whereas they were more heavily infected by the oomycete pathogen *Aphanomyces euteiches* and showed more disease symptoms (Gibelin‐Viala et al., 2019). Together, the abovementioned findings suggest a role of symbiotic molecules in suppressing immune responses, but more studies are required to understand the complicated immunity‐symbiosis interplay.

## 4 MODULATION OF PLANT IMMUNITY BY NON‐INVASIVE BENEFICIAL MICROBES

**Figure 2:**
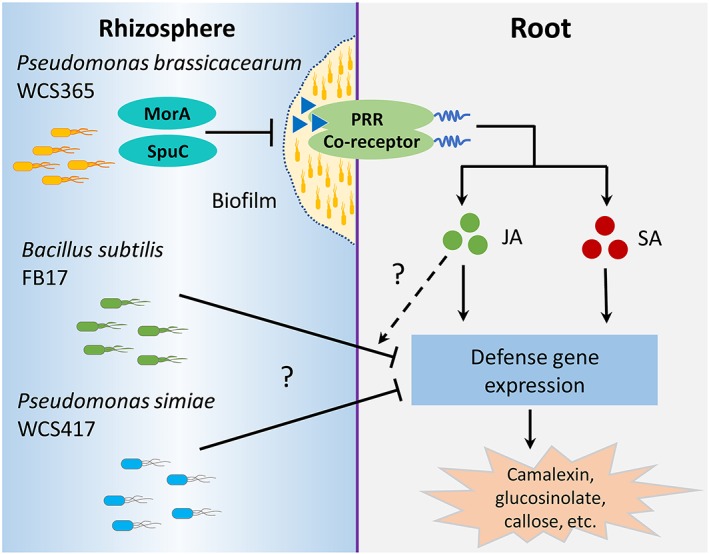
Schematic overview of root immune evasion and suppression mediated by non‐invasive beneficial microbes as described in the main text. Beneficial microbes living in rhizosphere can evade or suppress root immunity, suggesting that this is a useful trait for rhizosphere inhabitants [Colour figure can be viewed at http://wileyonlinelibrary.com]

Most of the abovementioned examples describe immune modulation strategies utilized by beneficial microbes that are invading host roots. Rhizobial cells differentiate intercellularly in legume nodules and also hyphae of mycorrhizal fungi penetrate host roots to form their symbiotic structures (Desbrosses & Stougaard, 2011; Garcia, Delaux, Cope, & Ane, 2015; Schmitz & Harrison, 2014). In nature, plants also form diverse beneficial associations with microbes that colonize the rhizosphere and promote plant growth or help the plant cope with adverse (a)biotic conditions (Bakker, Pieterse, de Jonge, & Berendsen, 2018; Berendsen et al., 2012). The rhizosphere is generally defined as the thin layer of soil at the root‐soil interface that is strongly influenced by root exudates (Bakker, Berendsen, Doornbos, Wintermans, & Pieterse, 2013), and thus, it can be debated to what extent the plant immune system can respond to mutualists living in this root exterior. It was shown that the root PRR gene *FLS2* displays a tissue‐ and cell type‐specific higher expression level at bacterial infection sites and at the inner cellular layers of *Arabidopsis* roots (Beck et al., 2014). Moreover, immune responses of the root pericycle were found to be stronger upon MAMP perception than those of other tissues (Wyrsch et al., 2015). These studies suggest that plants desensitize their root immune system at the outer cell layers of the root to prevent over‐responsiveness to the microbe‐rich soil environment. A non‐invasive lifestyle of certain beneficial microbes may thus prevent strong activation of plant immune responses. However, beneficial rhizosphere inhabitants, such as PGPR WCS417 and *B. subtilis* FB17, can actively suppress root immune responses (Lakshmanan et al., 2012; Millet et al., 2010; Stringlis, Proietti, et al., 2018; Figure 2). In *Arabidopsis* roots, both heat‐killed WCS417 cells and the WCS417 flg22 peptide can activate immune responses to the same extent as flg22 from the pathogen *P. aeruginosa* (Millet et al., 2010; Stringlis, Proietti, et al., 2018). Interestingly, an expression of more than 50% of the root immune‐responsive genes triggered by flg22 was repressed by live WCS417 cells (Stringlis, Proietti, et al., 2018). Recent evidence suggests that beneficial *Pseudomonas* spp. suppress flg22‐induced root immunity by producing organic acids that lower the environmental pH (Yu et al., 2019). *B. subtilis* FB17 also suppresses flg22‐induced immune responses in *Arabidopsis* roots but does this in a JA‐dependent manner (Lakshmanan et al., 2012; Figure 2). Moreover, Liu et al. (2018) identified 231 genes of the plant‐beneficial bacterium *Pseudomonas brassicacearum* WCS365 by high‐throughput transposon sequencing that confer increased bacterial fitness in the rhizosphere of wild‐type plants compared with the rhizospheres of immunocompromised plants. Clean deletion mutants that were generated for two of these genes, *morA* and *spuC*, also induced MTI in *Arabidopsis* roots. Both genes seemed to prevent intensive biofilm formation on roots, thereby preventing strong recognition and evading defence activation (Liu et al., 2018; Figure 2). These findings indicate that the root immune system actively influences microbes in the rhizosphere (Figure 2). In this light, Lebeis et al. (2015) found that *Arabidopsis* mutants, in which SA‐dependent defence signalling was disrupted, have distinct root microbiomes, suggesting that the immune system gates access and determines which microbes can colonize the roots. However, they found that SA‐dependent signalling primarily modulates the composition of the endophytic root microbiome, whereas the rhizosphere microbiome was less affected (Lebeis et al., 2015). Also, an assessment of wheat microbiomes after exogenous JA application demonstrated that JA signalling affects microbiome assembly in a compartment‐specific manner and that the endophytic root microbiome is mostly affected (Liu, Carvalhais, Schenk, & Dennis, 2017). In *Arabidopsis* on the other hand, JA signalling did affect the composition of *Arabidopsis* rhizosphere microbiomes, which could be associated to differences in root exudate profiles of JA signalling mutants compared with wild‐type plants (Carvalhais et al., 2015). Moreover, aboveground activation of the immune system by both microbial pathogens and insects has been demonstrated to result in alterations in rhizosphere microbiomes of several plant species (Berendsen et al., 2018; Dudenhöffer, Scheu, Jousset, & Cahill, 2016; Kong, Kim, Song, Lee, & Ryu, 2016; Yuan et al., 2018). Again, the microbiome alteration could be related to differential root exudation in response to activation of the immune system (Yuan et al., 2018). Recent studies identified coumarins (Stringlis et al., 2018; Voges, Bai, Schulze‐Lefert, & Sattely, 2019), benzoxazinoids (Hu et al., 2018), triterpenes (Huang et al., 2019), and camalexin (Koprivova et al., 2019) as chemical players that can shape the rhizosphere microbiome. Together, these findings suggest that although the gate keeping functions of the plant immune system might differ for different root compartments, the influence of the immune system does extend into the rhizosphere.

## 5 CONCLUDING REMARKS AND PROSPECTS

The plant immune system prevents most microbes from entering the root or reaching levels that are harmful to the plant. Irrespective of whether the association is harmful, neutral, or beneficial to the plant, microbes can evade and interfere with the plant immune system. To this end, members of the root microbiome possesses an immense repertoire of biosynthetic pathways that can produce bioactive compounds that interfere with host immunity (Stringlis, Zhang, et al., 2018). Plants will erect chemical and physical barriers that block the proliferation of those microbes inside the root that do not actively suppress this response. The root exterior is more open, and plants can only chemically steer rhizosphere microbiome composition. It is therefore likely that the influence of the plant in the rhizosphere declines gradually with increasing distance to the root as the concentration of root exudates decreases. Although it has been demonstrated that also some non‐invasive rhizosphere inhabitants possess the ability to suppress root immune responses, it is unknown whether this trait contributes to rhizosphere competence of microbes. Regardless, the rhizosphere is densely occupied by microbes and is likely a very MAMP‐rich environment. MAMP‐triggered activation of immunity leads to growth‐defence tradeoffs that hamper plant development (Huot, Yao, Montgomery, & He, 2014). It will therefore be intriguing to find out how plants prevent overstimulation of the plant immune system by MAMPs that are massively present around their roots.
